# Prospective multicentre study of host response signatures in neonatal sepsis in Sub Saharan Africa

**DOI:** 10.1038/s41598-022-25892-x

**Published:** 2022-12-12

**Authors:** Sem Ezinmegnon, Marine Mommert, Francois Bartolo, Gino Agbota, Sossou Darius, Valérie Briand, Marceline d’Almeida, Maroufou Jules Alao, Ida Dossou-Dagba, Achille Massougbodji, Ulrik Lausten-Thomsen, Alexandre Pachot, Laurence Vachot, Javier Yugueros-Marcos, Karen Brengel-Pesce, Nadine Fievet, Pierre Tissieres

**Affiliations:** 1 Open Innovation & Partnerships Lyon Sud (OI&P), bioMérieux, Marcy-l’Étoile, France; 2 Institute of Integrative Biology of the Cell (I2BC), CNRS, CEA, University Paris Saclay, Gif-sur-Yvette, France; 3 Soladis Statistics Inc., Lyon, France; 4 Institute of Research for Development (IRD), Université de Paris, MERIT, Paris, France; 5 Institut de Recherche Clinique du Bénin (IRCB), Abomey-Calavi, Benin; 6 INSERM, Institut de Recherche pour le Développement (IRD), UMR 1219, University of Bordeaux, Bordeaux, France; 7 Pediatric Department, National University Hospital Center (CNHU), Cotonou, Benin; 8 Pediatric Department, Mother and Child University and Hospital Center (CHUMEL), Cotonou, Benin; 9 Pediatric Department, Calavi Hospital, Abomey-Calavi, Benin; 10 Pediatric Intensive Care and Neonatal Medicine, AP-HP Paris Saclay University, Bicêtre Hospital, Le Kremlin-Bicêtre, France; 11 FHU Sepsis, AP-HP/Université Paris Saclay/Inserm, Le Kremlin-Bicêtre, France

**Keywords:** Biomarkers, Predictive markers, Health care, Paediatrics, Neonatal sepsis

## Abstract

Few biomarkers for sepsis diagnosis are commonly used in neonatal sepsis. While the role of host response is increasingly recognized in sepsis pathogenesis and prognosis, there is a need for evaluating new biomarkers targeting host response in regions where sepsis burden is high and medico-economic resources are scarce. The objective of the study is to evaluate diagnostic and prognostic accuracy of biomarkers of neonatal sepsis in Sub Saharan Africa. This prospective multicentre study included newborn infants delivered in the Abomey-Calavi region in South Benin and their follow-up from birth to 3 months of age. Accuracy of transcriptional (*CD74, CX3CR1*), proteic (PCT, IL-6, IL-10, IP-10) biomarkers and clinical characteristics to diagnose and prognose neonatal sepsis were measured. At delivery, cord blood from all consecutive newborns were sampled and analysed, and infants were followed for a 12 weeks’ period. Five hundred and eighty-one newborns were enrolled. One hundred and seventy-two newborns developed neonatal sepsis (29.6%) and death occurred in forty-nine infants (8.4%). Although PCT, IL-6 and IP-10 levels were independently associated with sepsis diagnosis, diagnostic accuracy of clinical variables combinations was similar to combinations with biomarkers and superior to biomarkers alone. Nonetheless, *CD74*, being the only biomarkers independently associated with mortality, showed elevated prognosis accuracy (AUC > 0.9) either alone or in combination with other biomarkers (eg.* CD74*/IP-10) or clinical criterion (eg. Apgar 1, birth weight). These results suggest that cord blood PCT had a low accuracy for diagnosing early onset neonatal sepsis in Sub Saharan African neonates, while association of clinical criterion showed to be more accurate than any biomarkers taken independently. At birth, *CD7*4, either associated with IP-10 or clinical criterion, had the best accuracy in prognosing sepsis mortality.

*Trial registration* Clinicaltrial.gov registration number: NCT03780712. Registered 19 December 2018. Retrospectively registered.

## Introduction

Neonatal sepsis remains a major societal and economic burden in low- and middle-income countries (LMIC). The Institute for Health Metrics and Evaluation estimates that in 2017, 200,000 newborns deaths occurred for every 2 million cases of sepsis in sub-Saharan Africa^1^. Early diagnosis of newborns with sepsis can reduce mortality and morbidity rates^2^. Unfortunately, the limited specificity of clinical signs, the lack of appropriate biological testing and the outrageous cost of sepsis treatment compared to national medium income in LMIC hamper most initiatives to reduce neonatal and pediatric mortality^3^. Difficulty in diagnosing sepsis in neonates is related to the conjunction of multiple variables, such as maternal health, pregnancy conditions, gestational age at delivery, newborn immune function, timing of infection and pathogens detection, as well as socio-economic environments and access to healthcare as seen in resources limited settings (RLS)^4^.

At birth, the immune defences go through rapid development and strengthen during the first years of life^5^. Studies have shown that the increased risk of early and late neonatal infections observed in both term and preterm infants are related to impaired innate immune function^6–8^. This innate immaturity is characterized by altered cytokines production and decreased function of antigen-presenting cells^9,10^. Blood culture remains the gold standard for the diagnosis of neonatal sepsis, but beside delayed results and low sensitivity, cost and access to those techniques in RLS limit its use^11,12^. Other tests routinely used for the diagnosis of neonatal sepsis, such as C-reactive protein (CRP) and blood count (total and differential white blood cell counts, absolute and immature neutrophil counts, and the ratio of immature to total neutrophils), are not unanimously recognized as good biomarkers of neonatal sepsis, respectively having a low specificity and lack of sensitiveness^13–16^. Additionally, repeated measurements are necessary to reach sufficient sensitivity limiting its use outside clinical bundles^15^. Procalcitonin (PCT) has been suggested to be a good marker for severe bacterial infection in newborn^17–19^. Several studies have shown that some pro-inflammatory (IL-6, IP-10) and anti-inflammatory (IL-10) cytokines can be used for the diagnosis of bacterial neonatal infection^20–24^. However, those cytokines profiles may be influeneced by gestational age, and time of onset of infection in newborn^21,25^. Based on a microarray study, a panel of genes has been identified in critically ill septic patients whose expression on peripheral blood could effectively help stratifying patients at increased risk of secondary infection and/or death^26,27^. Among these genes, *CD74* and *CX3CR1* were shown to be associated with mortality and/or the occurrence of secondary infections^28–30^. Low expression of *CX3CR1* is associated with mortality and immunosuppression in adult patients with septic shock^28–30^. Similarly, low expression of *CD74* is associated with mortality in adult patients with septic shock while increasing expression of *CD74* in the first days of intensive care is associated with the occurrence of secondary infections^29,31^. There are no data on value of *CD74* and *CX3CR1* for the prognosis or diagnosis of neonatal sepsis, which hamper current use of these biomarkers in neonates and children^15,16,32,33^.

In this study, we evaluated the diagnostic and prognostic accuracy of transcriptional (*CX3CR1, CD74*) and proteic (IL6, IL10, IP-10, PCT) biomarkers and their association with clinics and maternal risk factors for neonatal sepsis. In addition, we measured the reference range of those biomarkers during the first 3 months of life.

## Results

### Cohort description

**Figure 1:**
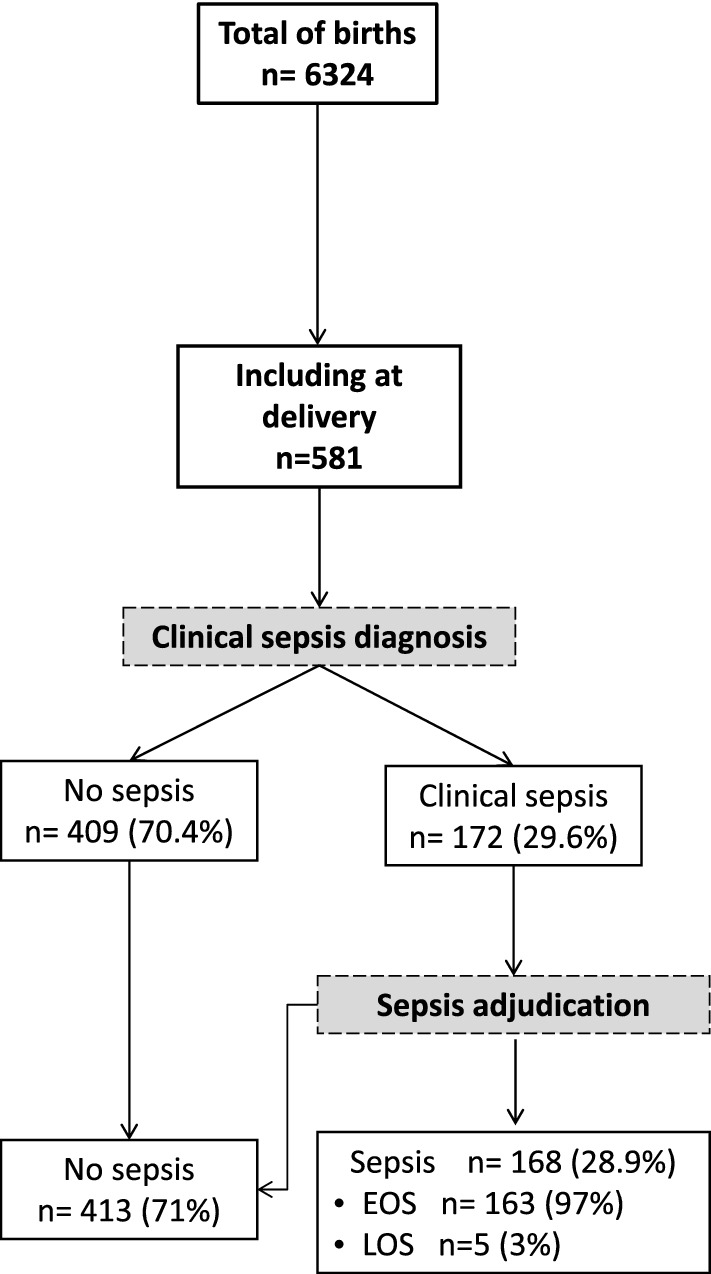
Flow diagram of study population. Recruitment and flow of newborn with risk of neonatal sepsis. At delivery, clinical sepsis diagnosis was established by the local pediatricians based on the child clinical examination and initial workup including haemogram, C-reactive protein (CRP) and microbiological cultures (blood, cerebral fluids and urine). All newborns with a clinical sepsis were subsequently adjudicated by two independent pediatricians and sorted into ‘LOS’ and ‘EOS’ (Figure drawn using Microsoft PowerPoint 2016, Redmond, CA).

Between April 17, 2016 and March 12, 2018, a total of 581 newborns were enrolled, 420 (72.3%) in the hospital arm and 161 (27.7%) in the sub-urban arm. Patients’ characteristics are displayed in Table 1. Median gestational age (GA) was 38.4 weeks (95% confidence interval [CI] 35.5–40 weeks), with a median birth weight of 2816 g (95% CI 2400–3150 g). One hundred and eighty-four (31.6%) infants were born prematurely with thirty-four (5.8%) being less than 32 weeks of GA. More preterm infants were delivered in the hospital arm than in the sub-urban arm (41.2% vs. 6.2%, *p* < *0.0001*) and had a lower birth weight (2700 g vs. 3006 g, *p* < *0.001*). Forty-five multiple gestations (40 twins, 5 triplicate) happened. Gestational malaria occurred in forty-three mothers (8.1%), affecting 49 infants (8.4%). Intermittent preventive treatment of malaria was performed in 470/531 (88.5%) of all mothers. Although methodologically considered as an exposure, the low gestational malaria occurrence did not justified a subgroup analysis. According to the study design, main differences between hospital and suburban arms were related to the perinatal risks with more premature infants and maternal risk factors of infection in the hospital arm. In total, 432/581 (74.3%) babies were born from mothers with maternal risk factors of infection (386/531, 72.7%). One hundred and seventy-two infants (29.6%) developed a clinical sepsis according to the definition. All occurred in the hospital arm. Among these, adjudication confirmed 168 cases (97.7%) (Fig. 1). All but five clinical sepsis were EONS. In the hospital arm, 307/420 infants were hospitalized following birth, the remaining with uneventful delivery and normal perinatal adaptation were discharged. Only one infant from the sub-urban arm was hospitalized for prenatally undiagnosed omphalocele. The median hospital stay was 4 days (95% CI 2–7 days). The global mortality was 49/581 (8.4%) with a significant difference between hospital and sub-urban arms, respectively 11.4% (48/420) versus 0.62% (1/161) (*p* < *0.001*). Most death occurred in the first week of life (median 2 days) and were related to neonatal sepsis (n = 44) or prematurity complications (n = 5).

**Table 1:** Patient characteristics.

		Suburban	Hospital non sepsis	Hospital sepsis	All patients	*p-*value
		(n = 161)	(n = 248)	(n = 172)	(n = 581)	
Female n (%)		81 (50.3)	129 (52)	77 (44.7)	287 (49.4)	*0.33*
Birth weight (grams)		3006 [2800–3205]	2700 [2350–3100]	2600 [1755–3100]	2816 [2400–3150]	< *0.001*
**Gestational age (week)**		39.5 [38.4–40.3]	38.1 [35.3–39.6]	36.1 [33.3–39.2]	38.4 [35.5–40]	< *0.001*
	≤ 37 weeks (%)	10 (6.2)	88 (35.4)	86 (50)	184 (31.6)	
	≤ 32 weeks (%)	1 (0.6)	8 (3.2)	24 (14)	34 (5.8)	
	≤ 28 weeks (%)	0 (0)	0 (0)	1 (0.6)	1 (0.1)	
APGAR score (1 min)		9 [9–9]	9 [8, 9]	8 [6–9]	9 [8–9]	< *0.001*
APGAR score (5 min)		10 [10–10]	10 [9–10]	9 [8–10]	10 [9, 10]	< *0.001*
Haemoglobin (g/dL)		15 [13.4–16.4]	14.8 [13.1–16.1]	14.9 [13.5–16.2]	14.9 [13.3–16.3]	*0.49*
Hospitalization		1 (0.6)	136 (54.8)	171 (99.4)	308 (53)	< *0.001*
Hospitalization duration (Day)		0 [0]	3 [1–4]	5 [3–10]	4 [2–7]	*N.C*
Death		1 (0.6)	10 (4)	38 (22.1)	49(8.4)	< *0.001*
Timing of death (Day)		0 [0–0]	1 [0.2–2]	3 [1–8]	2 [1–8]	*N.C*
Twins		4(0.6)	23(4)	18(3.1)	45 (7.7)	

Maternal characteristics		n = 157	n = 222	n = 152	n = 532	
Gestate		3 [2–4]	2 [1–4]	3 [1–4]	3 [1–4]	*0.005*
**Maternal risk factors**		12 (7.4)	248 (100)	172 (100)	432 (74.3)	< *0.001*
	Fever at delivery	3 (1.8)	56 (22.5)	62 (36)	108 (20.3)	< *0.001*
	Abnormal amniotic fluid	0 (0)	151 (60.9)	81 (47.1)	226 (42.5)	< *0.001*
	PROM (> 18 h)	0 (0)	77 (31)	70 (40.7)	137 (25.8)	< *0.001*
	Chorioamnionitis	0 (0)	20 (8)	27 (15.7)	46 (8.6)	< *0.001*
Malaria at delivery		6 (3.7)	24 (9.7)	19 (11)	49 (8.4)	*0.03*
IPT		160 (99.4)	203 (81.8)	143 (83.1)	506 (87)	< *0.001*

Qualitative data are expressed as numbers and frequency and quantitative data are expressed as medians and IQR (inter-quartile range: [Q1–Q3]). Qualitative variables were compared using the Chi-squared test (or Fisher’s exact test for small expected numbers). Anova test (or the Kruskal–Wallis test when distribution was not normal or when homoscedasticity was rejected) was performed to compare groups (statistically significant test *p* ≤ *0.05*).

*PROM,* Premature Rupture of Membranes; *IPT,* Intermittent Preventive Treatment of malaria; *APGAR*, neonatal adaptation score.

Identification of microorganisms in neonatal sepsis was performed using conventional bacterial culture followed by Biofire® FilmArray® multiplexing panel testing. Results are described in Table 2. A total of 155 hemocultures were sampled, of which positive cultures was obtained in 59/155 (38.1%) samples. Standard microbial cultures identified 59 pathogens and 14 hemocultures showed multiple germs and were considered as contaminated. Fifty-six of the 155 hemocultures were positive using Biofire® FilmArray® (56/155, 36.1%), in which 91 pathogens were identified. Gram-negative bacteria represented the majority of pathogens (52.5% to 49.4% pending the microbiologic technique) with *Enterobacter cloacae* complex, *Klebsiella pneumonia*, *Serratia marcescens* and *Staphylococcus spp*. being the most frequent germs identified (Table 2). Additional Biofire® FilmArray® panels identified viral infections (including Norovirus GI/GII, Rotavirus A, Coronavirus OC43, human rhinovirus and Respiratory Syncytial Virus) in 23 samples with negative standard cultures. Out of the 29 cerebrospinal fluid samples, one was positive for *Hemophilus influenzae*.

**Table 2:** Microbiology investigation results.

Pathogens	Standard culture	FilmArray®
**Gram-positive bacteria**	12 (20.3)	23 (25.3)
*Enterococcus*		1 (1.1)
Staphylococcus*	9 (15.2)	14 (15.4)
Staphylococcus aureus*		5 (5.5)
Streptococcus agalactiae (group B)*		1 (1.1)
streptococcus sp*	3 (5.1)	2 (2.2)
Gram-negative bacteria	31 (52.5)	45 (49.4)
Acinetobacter sp*	1 (1.7)	2 (2.2)
*Campylobacter*		2 (2.2)
Escherichia coli*	4 (6.7)	6 (6.6)
*Enterobacter cloacae complex*	11 (18.6)	12 (13.2)
*Haemophilus influenzae*		1 (1.1)
Klebsiella pneumoniae*	6 (10.1)	9 (10)
*Moraxella sp*	1 (1.7)	
Serratia marcescens*	8 (13.6)	13 (14.2)
**Virus**		23 (25.3)
Norovirus GI/GII		1 (1.1)
Rotavirus A		1 (1.1)
Coronavirus OC43		2 (2.2)
Detected Human Rhinovirus/Enterovirus		12 (13.2)
Respiratory Syncytial Virus		7 (7.7)
**Other**	16 (27.1)	
Candida non albicans	1 (1.7)	
Bacteria of different types (probable contamination)	14 (23.7)	
Bacteria non-identified	1 (1.7)	
**Total**	59 (100)	91 (100)

Data are shown as number (%) of patients.

* Pathogens found in co-infection.

### Primary outcosme: sepsis diagnosis

**Figure 2:**
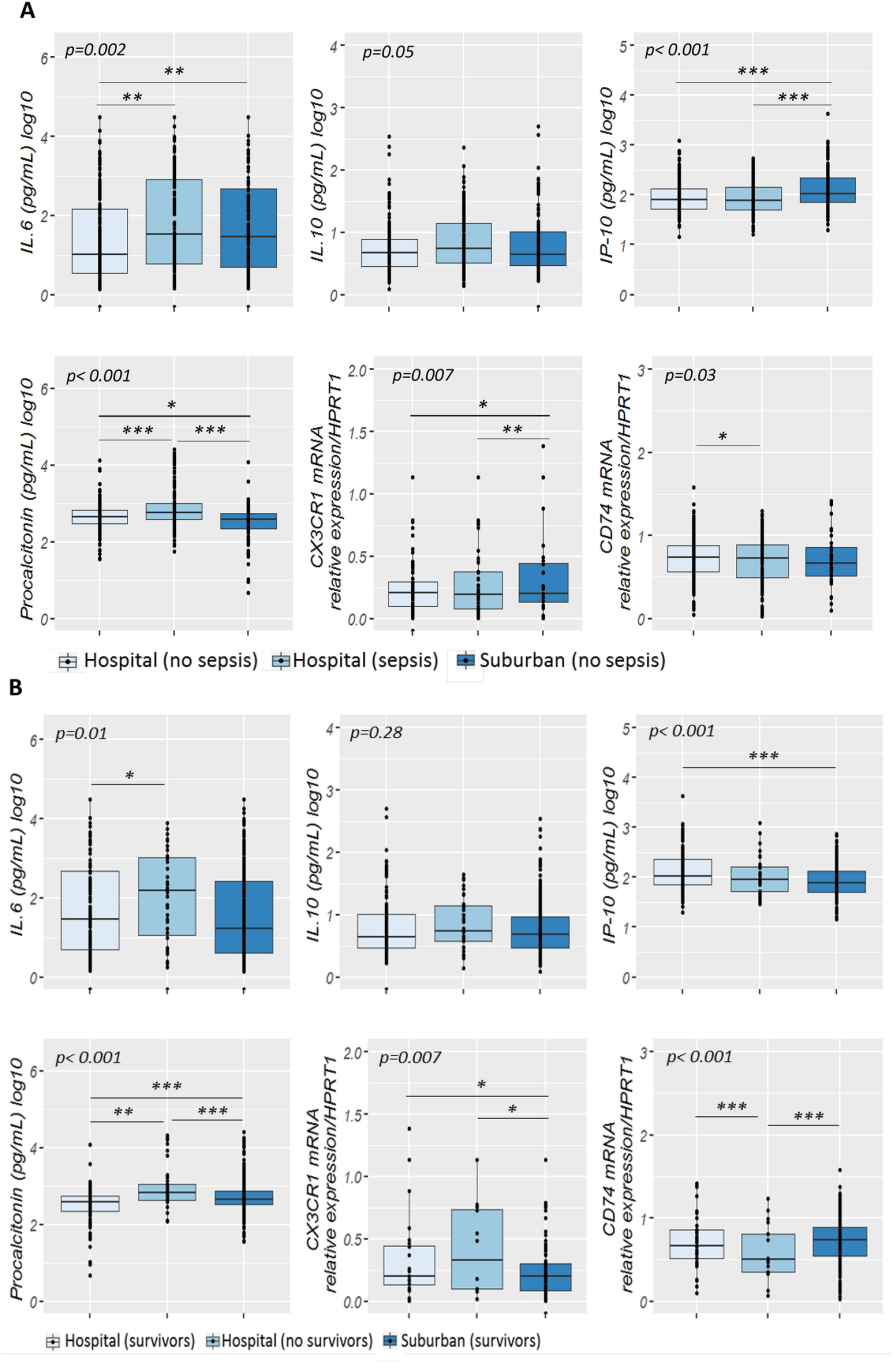
Cord blood biomarkers level in septic neonates. (**A**) Box-plot of transcriptional and protein biomarkers in clinical sepsis diagnosis. *CD74* and *CX3CR1* mRNA level were evaluated by RT-qPCR with ABI7500 fast and plasma PCT; IL6; IL10 and IP-10 concentrations were measured by multiplexed assay with the Ella platform in cord blood sample obtained from sepsis neonates and no sepsis neonates. Results are presented as box-plots as well as individual values in groups (hospital non-sepsis, hospital sepsis and non-sepsis suburban). Anova test (or the Kruskal-Wallis test when distribution was not normal or when homoscedasticity was rejected) was used to compare biomarkers. Data are shown as median, IQR with whiskers drawn within 1.5 IQR. A *p value* < 0.05 was considered as significant. A post hoc Student t test was performed to compare the groups against each other (*p ≤ *0.05*; **p < *0.01*; ***p < *0.001*). (**B**) Transcriptional and protein biomarkers expression in sepsis non survivors neonates and survivors neonates. *CD74* and *CX3CR1* mRNA level were evaluated by RT-qPCR with ABI7500 fast and plasma PCT; IL6; IL10 and IP-10 concentrations were measured by multiplexed assay with the Ella platform in cord blood sample obtained from non-surviving neonates and surviving neonates. Results are presented as box-plots as well as individual values in groups (hospital survivors, hospital non-survivors and suburban survivors). Anova test (or the Kruskal-Wallis test when distribution was not normal or when homoscedasticity was rejected) was used to compare biomarkers levels in suburban survivors, hospital non survivors and hospital survivor groups (statistically significant test *p* ≤ *0.05*). Data are shown as median, IQR with whiskers drawn within 1.5 IQR. A post hoc Student t test was performed to compare the groups against each other (*p ≤ *0.05*; **p < *0.01*; ***p < *0.001*).

**Figure 3:**
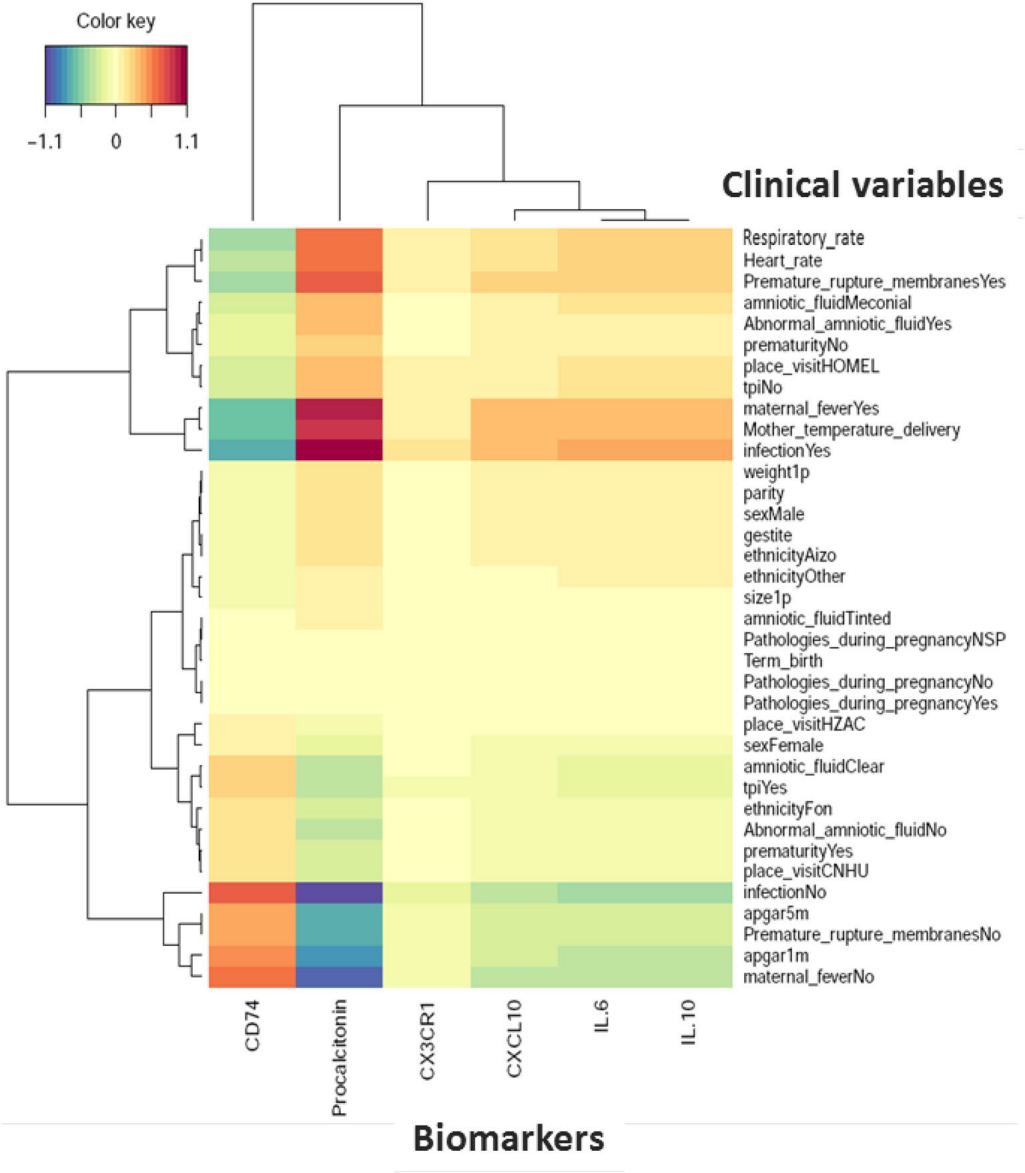
Biomarkers and clinical variables correlation in neonatal clinical sepsis diagnosis. Heatmap of correlation matrix (Partial Last Squares Regression) to predict biomarkers from Clinical Variables. The rows and columns correspond to clinical variables and biomarkers. Respectively positive correlations are red and negative correlations are blue. The figure shows only variables with covariances greater than max (covariance)/2 (Figure drawn using Rstudio, version2021.9.1.372, http://wwwrstudio.com, Boston MA).

Cord blood levels of studied biomarkers are displayed in Table 3. Procalcitonin level was significantly higher in infants developing neonatal sepsis compared to both sub-urban arm and hospital healthy infants (Fig. 2A). A significant difference in cord blood from non-septic neonates between both arms was observed. Diagnosis accuracy of different clinical variables and biomarkers alone and in association were tested in three models (Table 4). Model 1 encompassed patients from the hospital arm representing high-risk pregnancies. The model 2 compared hospital septic patients to all non-septic patients (hospital and suburban arm). The model 3 compared clinical sepsis with positive blood culture to all non-septic patients. Globally, none of tested biomarkers showed accurate diagnosis capacity. Combination of biomarkers with or without clinical criterion had similar diagnostic accuracy than combination of clinical criterion, irrespective of the model. Multivariate analysis including non-redundant clinical criterion and all biomarkers (Table 5), identified PCT (aOR 1.21, 95% CI 1.02–1.43, *p* = *0.027*), IL-6 (aOR 1.13, 95% CI 1.00–1.27, *p* = *0.043*), IP-10 (aOR 0.82, 95% CI 0.72–0.93, *p* = *0.002*) as well as Apgar score at 1 min > 7 (aOR 0.18, 95% CI 0.08–0.40, *p* < *0.001*), and maternal fever (aOR 2.8, 95% CI 1.58–5.03, *p* < *0.001*) as independently associated with sepsis diagnosis (model 2), with clinical criterion being the most strongly associated items with sepsis diagnosis. PCT cut-off value for a 96% diagnostic sensitivity (6% specificity) was 0.4 µg/mL similar to what has been published previously (Supplemental Table 1). Nevertheless, in the high-risk patients model 1, *CD74* to IP-10 ratio had the best diagnostic AUC (0.77, 95%CI 0.72–0.81). *CD74*/IP-10 ratio value of 0.44 (Youden index 0.21) had a sensitivity of 73% and specificity of 25% for clinical sepsis diagnosis. Correlation between biomarkers and clinical variables showed that *CX3CR1*, IP-10, IL-6 and IL-10 are poorly correlated with clinical variables (Fig. 3). However, *CD74* and PCT were strongly correlated (correlation cut-off < − 0.5 or > 0.5) with premature rupture of membrane (PROM), maternal fever, Apgar score, heart and respiratory rate (Fig. 3).

**Table 3:** Cord blood biomarkers level in neonatal sepsis.

Clinical sepsis					
	Hospital-Non sepsis (n = 248)	Hospital-sepsis (n = 167)	*p*-value *	Suburban-Non sepsis (n = 161)	*p*-value**
*CD74 *^*¶*^	5.2 [3.1–7.1]	4.7 [2–7.5]	*0.01*	4.6 [3.2–7.2]	*0.03*
*CX3CR1 *^*¶*^	0.8 [0.5–1.3]	0.8 [0.4–1.5]	*0.31*	1 [0.8–1.6]	*0.007*
IP-10 [pg/ml]	76.8 [49–134.6]	81.3 [56.9–143.1]	*0.51*	105 [72.6–223]	< *0.001*
IL-10 [pg/ml]	4.8 [2.9–8]	5.3 [3–13.5]	*0.01*	4.3 [2.9–10.2]	*0.05*
IL-6 [pg/ml]	7.3 [2.7–118.5]	29.9 [5.6–661.5]	< *0.001*	23.1 [4.5–468.5]	*0.002*
PCT [pg/ml]	458.3 [320.4–716.4]	506.6 [358.5–987.3]	< *0.001*	383 [225.7–543.7]	< *0.001*

Clinical Sepsis Adjudication					
	Hospital-Non sepsis (n = 252)	Hospital-sepsis (n = 163)	*p*-value*	Suburban-Non sepsis (n = 161)	*p*-value**
*CD74 *^*¶*^	5.3 [3.3–7.3]	4.6 [1.8–6.8]	*0.01*	4.6 [3.2–7.2]	0.23
*CX3CR *^*¶*^*1*	0.8 [0.5–1.4]	0.7 [0.4–1.3]	*0.37*	1 [0.8–1.6]	0.015
IP-10 [pg/ml]	79.5 [52.2–134.6]	78.5 [49.8–142.8]	*0.51*	105 [72.6–223]	< 0.001
IL-10 [pg/ml]	4.7 [2.8–7.7]	5.5 [3.2–14]	*0.03*	4.3 [2.9–10.2]	0.30
IL-6 [pg/ml]	8.3 [2.8–124.7]	26.8 [4.6–661.5]	< *0.001*	23.1 [4.5–468.5]	< 0.001
PCT [pg/ml]	443.8 [304.3–670.2]	583.5 [384.5–1022.7]	< *0.001*	383 [225.7–543.7]	< 0.001

Sepsis prognostic					
	Survivors hospital (n = 368)	Non survivors hospital (n = 47)	*p*-value*	Suburban Survivors (n = 160)	*p*-value**
*CD74 *^*¶*^	5.3 [3.2–7.5]	0.80 [0.3–3]	< *0.001*	4.5 [3.2–7.18]	< *0.001*
*CX3CR1 *^*¶*^	0.81 [0.4–1.4]	0.70 [0.3–1.2]	*0.23*	1 [0.7–1.59]	*0.12*
IP-10 [pg/ml]	77.0 [52–132]	87.0 [51.8–163.7]	*0.20*	104.9 [70.8–214.4]	*0.73*
IL-10 [pg/ml]	5.0 [3–9]	5.7 [3.8–14.1]	*0.10*	4.33 [2.94—10.2]	*0.10*
IL-6 [pg/ml]	14.0 [4–213]	147.0 [8.7–1034.2]	*0.002*	26.71 [4.5–186.7]	*0.005*
PCT [pg/ml]	463.0 [338–760]	701.0 [424–1127]	*0.001*	383.8 [227.8–557.5]	< *0.001*

Biomarkers are expressed as median and inter-quartile range (IQR: Q1–Q3).

^*¶*^* CD74* and *CX3CR1* are expressed as relative expression to *HPRT1.*

*Student’s t-test (or the Mann–Whitney t-test when distribution was not normal or Welch test when homoscedasticity was rejected) were performed to compare two hospital arms groups.

**Anova test was performed to compare biomarkers levels between the three groups.

*p* value was considered significant ≤ *0.05.*

*CD74*, HLA class II histocompatibility antigen gamma chain; *CX3CR1;* CX3C chemokine receptor 1; *IL*, Interleukin; PCT, procalcitonin.

**Table 4:** Area under the receiver operating curve of biomarkers and clinical variables combinations for the diagnosis of sepsis.

Variables	Model 1* AUC (95% CI)	Model 2** AUC (95% CI)	Model 3*** AUC (95% CI)
4 CV	0.71 (0.65–0.76)	0.88 (0.83–0.93)	0.77 (0.65–0.89)
5 CV	0.72 (0.67–0.77)	0.86 (0.82–0.91)	0.76 (0.66–0.85)
12CV	0.71 (0.66–0.77)	0.88 (0.83–0.93)	0.78 (0.68–0.89)
17 CV	0.74 (0.68–0.78)	0.74 (0.67–0.80)	0.76 (0.66–0.86)
3 Biomarkers + 4 CV	0.72 (0.66–0.77)	0.78 (0.72–0.84)	0.76 (0.65–0.86)
3 Biomarkers + 5 CV	0.73 (0.67–0.78)	0.75 (0.69–0.81)	0.75 (0.65–0.86)
6 Biomarkers + 17 CV	0.75 (0.69–0.79)	0.76 (0.69–0.82)	0.77 (0.68–0.87)
2 Biomarkers + 5 CV	0.71 (0.65–0.76)	0.76 (0.70–0.82)	0.75 (0.63–0.86)
1 Biomarkers + 5 CV	0.71 (0.65–0.76)	0.76 (0.69–0.82)	0.76 (0.65–0.87)
All Biomarkers	0.68 (0.62–0.73)	0.72 (0.65–0.79)	0.77 (0.66–0.88)
All proteins	0.65 (0.64–0.65)	0.71 (0.64–0.78)	0.79 (0.68–0.89)
*CX3CR1 & CD74*	0.61 (0.60–0.62)	0.61 (0.54–0.69)	0.63 (0.50–0.75)
*CD74*	0.59 (0.53–0.65)	0.58 (0.52–0.64)	0.55 (0.45–0.65)
*CX3CR1*	0.54 (0.47–0.59)	0.56 (0.49–0.62)	0.54 (0.43–0.64)
IL-10	0.56 (0.49–0.61)	0.58 (0.52–0.64)	0.57 (0.48–0.67)
IL-6	0.51 (0.44–0.56)	0.62 (0.56–0.67)	0.66 (0.57–0.74)
IP-10	0.51 (0.45–0.57)	0.55 (0.50–0.61)	0.54 (0.45–0.63)
PCT	0.62 (0.56–0.67)	0.65 (0.59–0.70)	0.64 (0.55–0.73)
*CD74*/*CX3CR1*	NA	0.52 (0.45–0.58)	0.51 (0.41–0.61)
*CD74*/IP-10	0.77 (0.72–0.81)	0.54 (0.48–0.61)	0.54 (0.44–0.65)
*CD74*/IL-10	0.59 (0.53–0.65)	0.62 (0.55–0.68)	0.57 (0.46–0.67)
*CD74*/IL-6	NA	0.63 (0.57–0.70)	0.65 (0.55–0.75)
*CD74*/PCT	0.63 (0.57–0.69)	0.66 (0.59–0.72)	0.62 (0.52–0.72)
IL-10/IP-10	0.60 (0.53–0.65)	0.63 (0.58–0.69)	0.57 (0.48–0.66)
IL-6/IP-10	0.63 (0.55–0.67)	0.64 (0.59–0.70)	0.66 (0.57–0.74)
IL-6/IL-10	0.51 (0.44–0.57)	0.60 (0.55–0.66)	0.65 (0.57–0.73)
PCT/IL-10	0.55 (0.48–0.58)	0.56 (0.50–0.62)	0.57 (0.48–0.67)
PCT/IP-10	0.74 (0.68–0.79)	0.66 (0.61–0.71)	0.61 (0.52–0.69)
PCT/IL-6	NA	0.57 (0.51–0.62)	0.60 (0.51–0.69)

* MODEL 1: Hospital arm sepsis (n = 163) **vs** hospital arm no sepsis (n = 257).

** MODEL 2: Hospital arm sepsis (n = 163) **vs** no sepsis (suburban arm n = 47 & hospital arm n = 245).

*** MODEL 3: Hospital arm sepsis with positive blood culture (n = 55) **vs** no sepsis (suburban arm n = 47 & hospital arm non sepsis n = 245).

*CD74*, HLA class II histocompatibility antigen gamma chain; *CX3CR1;* CX3C chemokine receptor 1; *IL*, Interleukin; PCT, procalcitonin.

Clinical variables (CV) combination legend:

4 CV: Apgar 1 m, location of consultation, height, birth temperature.

5 CV : Apgar 1 m, location of consultation, height, birth temperature, term of birth.

12 CV : Apgar 1 m, Apgar 5 m, chorioamnionitis, heart frequency, heart frequency1P, location of consultation, abnormal amniotic fluid, birth weight, sex, height, birth temperature, term of birth.

17 CV : Apgar 1 m, Apgar 5 m, maternal fever, heart frequency, heart frequency1P, gravidity, chorioamnionitis, abnormal amniotic fluid, location of consultation, amniotic fluid, birth weight, prematurity, premature membranes rupture, sex, height, birth temperature, term of birth.

3 Biomarkers + 4 CV : *CD74*, IP-10, IL-10, Apgar 1 m, location of consultation, height, birth temperature.

3 Biomarkers + 5 CV : *CD74*, IP-10, IL,10, Apgar 1 m, location of consultation, height, birth temperature, term of birth.

6 Biomarkers + 17 CV : *CD74*, *CX3CR1*, IP-10, IL-10, IL-6, Procalcitonin, Apgar 1 m, Apgar 5 m, maternal fever, heart frequency, Heart frequency 1P, gravidity, chorioamnionitis, abnormal amniotic fluid, location of consultation, amniotic fluid, birth weight, prematurity, premature membranes rupture, sex, height, birth temperature, term of birth.

2 Biomarkers + 5 CV : IP-10, procalcitonin, Apgar 1 m, location of consultation, sex, height, birth temperature.

1 Biomarkers + 5 CV : Procalcitonin, Apgar 1 m, chorioamnionitis, location of consultation, height, birth temperature.

**Table 5:** Factors associated with clinical sepsis using univariate and multivariate logistic regression models.

Characteristics	Univariate		Multivariate	
	_crude_ OR [95% CI]	*p* value	_Adjusted_ OR [95% CI]	*p* value
Multi gravidity	1.21 [0.73;1.98]	0.461	–	
IPT	0.59 [0.29;1.20]	0.145	–	
Maternal fever	2.68 [1.64;4.39]	< 0.001	2.82 [1.58;5.03]	< 0.001
Maternal infection	2.61 [1.27;5.39]	0.009	–	
PROM	1.73 [1.09;2.76]	0.020	–	
Abnormal amniotic fluid	0.86 [0.55;1.34]	0.511	–	
Apgar at 1 min > 7	0.17 [0.08;0.33]	< 0.001	0.18 [0.08;0.40]	< 0.001
Apgar at 5 min > 7	0.18 [0.06;0.52]	0.001	–	
Length (*cm*)	0.86 [0.80;0.92]	< 0.001	0.84 [0.78;0.91]	< 0.001
Sex (Female)	0.79 [0.50;1.23]	0.291	–	
Preterm birth	2.53 [1.59;4.00]	< 0.001	–	
Low birthweight	2.28 [1.43;3.62]	< 0.001	–	
Tachycardia (> 160 bpm)	2.56 [1.31;5.01]	0.006	–	
*CD74 *^*¶*^	0.04 [0.00;0.54]	0.016	0.21 [0.01;5.15]	0.342
*CX3CR1 *^*¶*^	1.63 [0.50;5.33]	0.421	1.77 [0.28;11.35]	0.421
IP-10 (pg/ml)	0.90 [0.81;0.99]	0.033	0.82 [0.72;0.93]	0.002
IL-10 (pg/ml)	1.05 [0.61;1.81]	0.859	0.98 [0.33;2.84]	0.963
IL-6 (pg/ml)	1.11 [1.02;1.21]	0.016	1.13 [1.00;1.27]	0.043
PCT (pg/ml)	1.35 [1.11;1.64]	0.002	1.21 [1.02;1.43]	0.027

IPT, intermittent preventive treatment against malaria; *CD74*, HLA class II histocompatibility antigen gamma chain; *CX3CR1;* CX3C chemokine receptor 1; *IL*, Interleukin; PCT, procalcitonin.

^*¶*^* CD74* and *CX3CR1* are expressed as relative expression to *HPRT1.*

### Secondary outcome: mortality

At delivery, PCT, *CD74*, IL-6 and *CX3CR1* levels were significantly different between survivors and non survivors (Fig. 2B). Level of expression of *CD74* showed good prognosis accuracy in model 2 and 3, as well as being systematically retrieved in most combinational models (Table 6). Interestingly, best prognosis accuracy occurred with the combination of biomarkers and clinical items, with *CD74*, Apgar score at 1 min and birth weight having an AUC of 0.97 (95% CI 0.94–0.99). On multivariate analysis (Table 7), *CD74* was the only biomarkers independently associated with mortality (aOR 0.69, 95% CI 0.57–0.83, *p* < 0.001) along with intrauterine growth retardation (aOR 4.32, 95% CI 1.07–17.34, *p* = *0.039*), and skin motteling (aOR 55.5, 95% CI 3.67838.97, *p* = *0.003*).

**Table 6:** Area under the receiver operating curve for the prognosis of neonatal clinical sepsis.

Variables	MODEL 1^a^ AUC (95% CI)	MODEL 2 ^b^ AUC (95% CI)	MODEL 3 ^c^ AUC (95% CI)
3 CV *	NA	0.88 (0.81–0.95)	0.90 (0.79–1.01)
3 CV **	0.81 (0.74–0.89)	0.89 (0.83–0.95)	0.89 (0.73–1.05)
4 CV	0.82 (0.74–0.91)	0.89 (0.82–0.95)	0.90 (0.78–1.03)
6 CV	0.83 (0.75–0.90)	0.90 (0.81–0.99)	0.92 (0.82–1.02)
9 CV	0.85 (0.78–0.92)	0.90 (0.81–0.99)	0.88 (0.77–0.98)
11 CV	0.86 (0.78–0.92)	0.89 (0.83–0.96)	0.88 (0.68–1.08)
3 Biomarkers + 6 CV	0.91 (0.86–0.95)	0.95 (0.92–0.98)	0.92 (0.83–1.00)
1 Biomarkers + 4 CV	0.91 (0.85–0.96)	0.95 (0.92–0.99)	0.89 (0.81–0.98)
1 Biomarkers + 6 CV	0.91 (0.85–0.96)	0.96 (0.94–0.99)	0.95 (0.87–1.02)
2 Biomarkers + 3 CV	0.91 (0.86–0.96)	0.95 (0.90–0.99)	0.90 (0.78–1.02)
6 Biomarkers + 16 CV	0.93 (0.89–0.97)	0.95 (0.92–0.98)	0.92 (0.82–1.02)
5 Biomarkers + 6 CV	0.92 (0.88–0.96)	0.95 (0.92–0.98)	0.94 (0.86–1.02)
3 Biomarkers + 4 CV	0.91 (0.86–0.96)	0.96 (0.94–0.98)	0.92 (0.83–1.01)
1 Biomarker + 2 CV	0.86 (0.79–0.92)	0.97 (0.94–0.99)	0.90 (0.79–1.02)
All biomarkers	0.83 (0.73–0.92)	0.84 (0.74–0.93)	0.84 (0.65–1.02)
All proteins	0.64 (0.63–0.65)	0.69 (0.59–0.80)	0.82 (0.69–0.95)
*CX3CR1 & CD74*	0.82 (0.75–0.89)	0.83 (0.74–0.92)	0.77 (0.60–0.94)
*CD74*	0.78 (0.68–0.87)	0.82 (0.73–0.91)	0.83 (0.68–0.98)
*CX3CR1*	0.51 (0.40–0.61)	0.56 (0.45- 0.68)	0.63 (0.42–0.84)
IL-10	0.79 (0.72–0.85)	0.58 (0.48–0.68)	0.73 (0.55–0.91)
IL-6	0.72 (0.65–0.80)	0.64 (0.55–0.73)	0.72 (0.57–0.87)
IP-10	0.54 (0.44–0.65)	0.54 (0.44–0.63)	0.73 (0.57–0.89)
PCT	0.62 (0.52–0.72)	0.68 (0.59–0.76)	0.73 (0.55–0.91)
*CD74*/*CX3CR1*	NA	0.74 (0.63–0.84)	0.64 (0.41–0.87)
*CD74*/IP-10	0.80 (0.71–0.88)	0.83 (0.75–0.92)	0.91 (0.85–0.97)
*CD74*/IL-10	0.74 (064–0.85)	0.79 (0.69–0.88)	0.86 (0.77–0.95)
*CD74*/IL-6	NA	0.75 (0.66–0.84)	0.80 (0.70–0.90)
*CD74*/PCT	0.87 (0.79–0.94)	0.82 (0.73–0.91)	0.87 (0.79–0.96)
IL-10/IP-10	0.67 (0.58–0.75)	0.49 (0.39–0.58)	0.56 (0.33–0.79)
IL-6/IP-10	0.66 (0.58–0.74)	0.64 (0.55–0.73)	0.67 (0.51–0.84)
IL-6/IL-10	0.56 (0.45–0.66)	0.63 (0.54–0.73)	0.67 (0.51–0.83)
PCT/IL-10	0.55 (0.44–0.66)	0.58 (0.48–0.68)	0.54 (0.27–0.80)
PCT/IL-6	NA	0.60 (0.51–0.69)	0.66 (0.51–0.81)
PCT/IP-10	0.56 (0.46–0.66)	0.63 (0.53–0.72)	0.60 (0.43–0.78)

^a^Hospital arm survivors (n = 372) vs hospital arm no survivors (n = 48).

^b^Survivors suburban arm (n = 47) and hospital arm (n = 368)) versus Hospital arm no survivors (n = 47).

^c^Survivors (n = 399) vs Hospital arm with positive blood culture sepsis no survivors ( n = 10).

*CD74*, HLA class II histocompatibility antigen gamma chain; *CX3CR1;* CX3C chemokine receptor 1; *IL*, Interleukin; PCT, procalcitonin.

Clinical variables combination legend:

3 CV ^¶^: Birth weight, Height, term of birth.

3 CV ^¶¶^: Apgar 1 m, birth weight, sex.

4 CV : Apgar 5 m, skin color, birth weight, term of birth.

6 CV : Apgar 5 m, gravidity, abnormal amniotic fluid, birth weight, height, term of birth.

9 CV : Apgar 1 m, Apgar 5 m, gravidity, abnormal amniotic fluid, chorioamnionitis, birth weight, sex, height, term of birth.

11 VC : Apgar 1 m, Apgar 5 m, skin color, gravidity, location of consultation, abnormal amniotic fluid, parity, birth weight, sex, height, term of birth.

3 Biomarkers + 6 CV : *CD74*, *CX3CR1*, IP-10, Apgar 5 m, ethnicity, abnormal amniotic fluid, birth weight, height, term of birth.

1 Biomarkers + 4 CV : *CD74*, Apgar 5 m, skin color, location of consultation, term of birth.

1 Biomarkers + 6 CV : *CD74*, Apgar 5 m, skin color, location of consultation, chorioamnionitis, birth weight, term of birth.

2 Biomarkers + 3 CV : *CD74*, IL-10, amniotic fluid, term of birth, intermittent preventive treatment.

6 Biomarkers + 16 CV : *CD74*, *CX3CR1*, IP-10, IL-10, IL-6, procalcitonin, Apgar 1 m, Apgar 5 m, skin color, ethnicity, gravidity, abnormal amniotic fluid, location of consultation, amniotic fluid, parity, chorioamnionitis, birth weight, prematurity, sex, height, term of birth, intermittent preventive treatment;

5 Biomarkers + 6 CV : *CD74*, *CX3CR1*, IL-10, IL-6, procalcitonin, Apgar 1 m, Apgar 5 m, location of consultation, birth weight, height, term of birth.

3 Biomarkers + 4 CV : *CD74*, IP-10, IL-10, Apgar 1 m, birth weight, height, term of birth.

1 Biomarker + 2 CV : *CD74*, Apgar 1 m, birth weight.

**Table 7:** Factors associated with infant’s death using univariate and multivariate logistic regression models.

Characteristics	Univariate		Multivariate	
	_Crude_ OR [95% CI]	*p* value	_Adjusted_ OR [95% CI]	*p* value
Gravidity	1.42 [0.59;3.41]	*0.437*	–	
IPT	0.46 [0.17;1.21]	*0.116*	–	
Maternal fever	1.52 [0.70;3.31]	*0.293*	–	
Maternal infection	1.34 [0.44;4.08]	*0.609*	–	
PROM	1.27 [0.59;2.72]	*0.539*	–	
Abnormal amniotic fluid	0.38 [0.17;0.83]	*0.015*	–	
Apgar at 1 min > 7	0.26 [0.12;0.59]	*0.001*	–	
Apgar at 5 min > 7	0.15 [0.06;0.42]	< *0.001*	–	
Skin mottling	43.6 [8.58;221.56]	< *0.001*	55.5 [3.67;838.97]	*0.004*
Sex	0.67 [0.32;1.41]	*0.290*	–	
Preterm birth	5.87 [2.53;13.58]	< *0.001*	–	
Low birthweight	6.43 [2.77;14.91]	< *0.001*	4.32 [1.11;16.80]	*0.035*
Tachycardia (> 160 bpm)	2.43 [0.97;6.10]	*0.058*	–	
*CD74 *^***¶***^	0.67 [0.56;0.80]	< *0.001*	0.72 [0.61;0.86]	< *0.001*
*CX3CR1 *^***¶***^	0.82 [0.25;2.64]	*0.740*	0.94 [0.20;4.32]	*0.934*
CXCL10 (pg/ml)	1.09 [0.99;1.19]	*0.074*	1.12 [0.99;1.27]	*0.071*
IL10 (pg/ml)	0.95 [0.35;2.56]	*0.920*	0.38 [0.03;4.16]	*0.429*
IL6 (pg/ml)	1.03 [0.95;1.12]	*0.421*	0.96 [0.83;1.10]	*0.421*
PCT (pg/ml)	1.11 [1.02;1.20]	*0.015*	1.10 [0.98;1.23]	*0.076*

IPT, intermittent preventive treatment against malaria; *CD74*, HLA class II histocompatibility antigen gamma chain; *CX3CR1;* CX3C chemokine receptor 1; *IL*, Interleukin; PCT, procalcitonin.

^*¶*^* CD74* and *CX3CR1* are expressed as relative expression to *HPRT1.*

### Biomarkers reference range in the first 12 weeks of life

**Figure 4:**
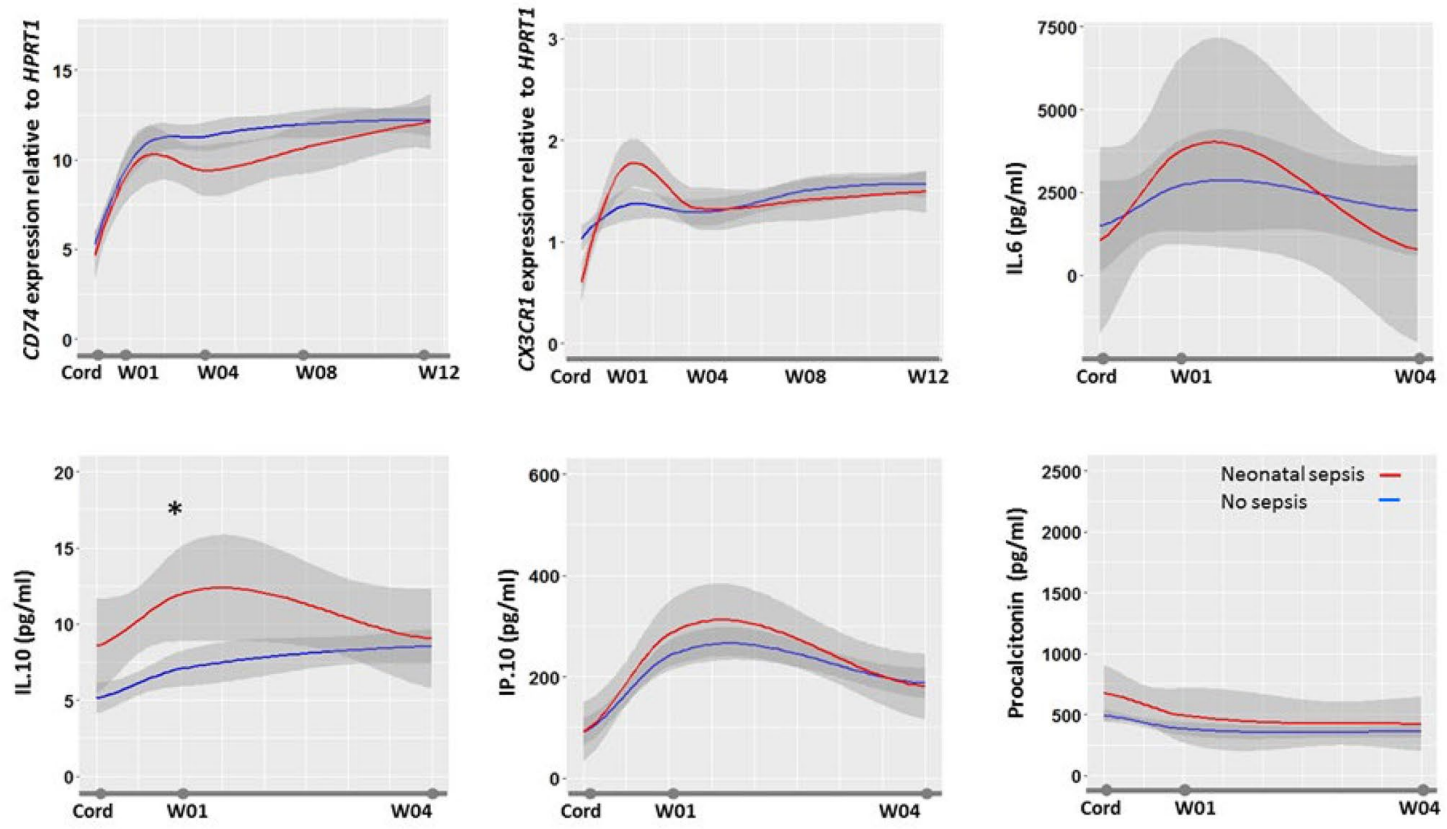
Biomarkers kinetics during 3 months of follow-up. The figure shows the biomarkers profile during the first 3 months of life in healthy infants with median value (in blue), 95%confidence interval (in gray), and median value in septic infants (in red). Biomarkers were measured at delivery in cord blood (Cord) and at first, 4, 8 and 12 weeks in peripheral blood. Data are expressed as medians. Anova test was used to compare biomarkers levels.

In healthy newborns, all biomarkers but PCT had a significant lower value on cord blood than in the first week following birth (Fig. 4). Whereas most biomarkers range did not vary between weeks 1 and 12, *CD74* progressively increased during the first 8 weeks of life. Following neonatal sepsis, at week 4, *CD74* was significantly lower in septic than in healthy newborns. We did not observe any significant differences in biomarker profiles between newborns exposed or not to gestational malaria.

## Discussion

This multicentre study performed in sub Saharan Africa showed that clinical criteria either related to the infant or the mother are superior to individual cord blood biomarkers^34^. Nonetheless, *CD74*/IP-10 ratio may prove some interest in a subgroup of high-risk patients (prematurity, multiple gestation, maternal infection). Although cord blood PCT value was shown to be independently associated with early-onset sepsis diagnosis, it has a poor accuracy (cutoff similar to previous studies 0.4 ng/mL). In contrast, cord-blood biomarkers especially when associated with clinical criterion was shown to be highly predictive of outcome. *CD74* was shown to be the only prognosis biomarkers independently associated with outcome and its association with Apgar at 1 min, intrauterine growth retardation having the highest prognosis accuracy.

The evaluation of clinical and biomarkers for neonatal sepsis diagnosis and prognosis in setting with high prevalence of sepsis and limited resources is bringing for the first-time detailed assessment of potential biomarkers utility and establishment of reference values in neglected pediatric populations. As such, this study is a radical breach in biomarkers development paradigms by re-focusing their evaluation in settings with high prevalence of neonatal sepsis and low ressources. Several large studies, occurring in setting with high resources and low sepsis prevalence, have suggested that cord blood PCT may be a robust biomarker for neonatal sepsis diagnosis, reporting elevated sensitivity and specificity for cut-off values ranging between 0.6 and 0.7 ng/mL^35–37^. These encouraging data were not confirmed by all. Few series in extreme premature infants and in setting where EONS prevalence was higher showed lower sensitivity and specificity ranging between 48.7 to 69% and 68.6 to 70%, respectively^38,39^. Our study confirms the inaccuracy of cord blood PCT in diagnosing neonatal sepsis. The lack of specificity that we observed may be related to the effect of maternal and neonatal medical or environmental conditions on PCT levels. In a large series involving 2′151 infants (26 with EONS), Joram et al*.* identified gestational age (28–32 weeks) and pH < 7.10 to be the only factors associated with increased PCT levels^36^. In our series, PCT was correlated with PROM, maternal fever, Apgar score, gestational age, infant heart and respiratory rate, suggesting that prenatal condition and newborn adaptation impact PCT level as displayed by a significant PCT level difference in healthy infants born from either the hospital (with maternal risk factors) or the sub-urban arm (no maternal risk factors).

Interestingly, in the sub-group of high-risk patients *CD74*/IP-10 ratio was shown for the first time to have a better accuracy for sepsis diagnosis than any studied biomarkers and clinical combinations. CD74, the invariant chain involved in MHC class II molecules transport, is identified as a prognosis marker in adult critically ill patients developing healthcare associated infections^31^. In our study, *CD74* expression alone has a low accuracy for neonatal sepsis diagnosis, but its association with IP-10, the interferon-γ inducible protein-10, emerges as a good combination of biomarker. IP-10 is associated with various inflammatory conditions such as autoimmune diseases, hemophagocytic syndromes and viral infections where its combination with phospholipase A2, or IL-10 were suggested for sepsis diagnosis^23,40–42^. Combination of *CD74* and IP-10, illustrating both antigen presentation ability and response to type II interferon stimulation, may be representative of both faces of neonatal immunological competency to infection. Ontogeny of antigen presentation in early life is thought to be a key factor in determining age-specific responses to microbes and other antigens. IP-10 production in infants reflects a consequent Th1 response and as such, it may represent in conjunction with a reduced *CD74* expression, despite IFNγ stimulation, a signature for severely dysregulated response associated with neonatal sepsis.

Although well recognized in cancer, few publications relate *CD74* utility in sepsis, none in children and neonates^43,44^. In contrast, in a study of critically ill septic adults, low expression of *CD74* was frequently shown to be independently associated with mortality^29^. whereas secondary infection occurrence was associated with increased *CD74* expression in the first day following admission for septic shock^41^. Our study further extend *CD74* prognosis utility in neonatal sepsis. *CD74* expression on cord blood of < 6.36–12.37 cutoff value was predictive of death in neonates with a sensitivity of 95–100% and a specificity of 10–36%, pending the model (Supplemental Table 1). Indirectly those results confirm those related to histocompatibility leukocytes antigen-DR expression on monocytes (mHLA-DR) that is known to be highly correlated (r = 0.87) with *CD74* expression[38]. Low mHLA-DR was shown to be associated with EONS in premature infants and mortality following LONS^45,46^. The underlying pathophysiologic rational behind the association of low CD74/low mHLA-DR in infants and sepsis mortality may be resumed by an altered antigen presentation, either acquired or secondary to a perinatal event. Primary defect or decreased expression of mHLA-DR was shown to be associated with prematurity and altered myeloid cells functionality in extremely immature infants^8,47^. Secondary decrease of mHLA-DR in response to diverse aggression is well recognized. Decrease in mHLA-DR expression was demonstrated following sepsis, and is recognized as one of the main immunological caracterization of post-infective immune failure^48–50^. As such, the prolonged alteration of mHLA-DR was shown to be associated with mortality in adults with sepsis^48,51–53^. Serial mHLA-DR kinetics in septic newborns is largely unknown although a rise in HLA-DR negative monocytes following severe sepsis is reported^54^. Interestingly, in our study, infants surviving sepsis showed a prolonged alteration of *CD74* expression up to 1 month following EONS (Fig. 4). Nevertheless, such prolonged post-sepsis reduction in *CD74* transcription, as well as reduced mHLA-DR has never been described in both infants and adults. Newborns are known to have a rapidly evolving immunity during the first months of life. Olin et al. demonstrated in a longitudinal analysis of infant’s immunity that a phenotypic convergence is occurring between term and preterm infants at 3 months of age, but in between environmental factors shape immune variation^55^. Interestingly they showed that drastic changes in cell composition and phenotypes, plasma protein concentrations occurred during the first few days of life bringing additional insights to the understanding of biomarkers kinetics and potential utility for diagnosing EONS. Accordingly, our study establishes the reference values of IL-6, IL-10, IP-10 and *CD74, CX3CR1* during the first 4 and 12 weeks of life, respectively. These are the first published set of reference values from newborn and infants living in Sub Saharan Africa. Extrapolation of these reference values to infants outside Africa may be acceptable. We showed that gestational malaria did not impact any biomarkers values making these reference values applicable in region with no endemic malaria. In addition, a recent study showed that age-related immune patterns was not different between Africa, North America, South America and Europe despite environmental variations^56^.

In addition to the forehead mentioned biomarkers limitations, additional should be acknowledged. Although intensive workup has been set to obtain systematic microbiologic documentation, it is plausible that model 3 (based on culture positive sepsis) may under-evaluate the number of positive cases considering residual technical and organizational issues. However, the consistency of the analysis along the three models suggest that the impact is minimal. The most challenging methodologic issue was the follow-up of the children up to 3 months of age. Local individuals serving as official community advisor, organized local health care centers, midwifes and dedicated physicians and nurses organized the systematic follow-up of all children. Community advisor allowed to have close and honest collaboration with local authorities (village chief) permitting to reassure and pursue the follow-up.

Those results may directly affect management of infants with EONS in LRS. First, our data emphasize the importance of clinical criteria for EONS diagnosis and therefore the importance of implementing clinical curriculum to train midwifes, nurses and physicians taking care of neonates. Second, structured perinatal track at a regional level may help identifying high-risk populations and optimizing resources allocation. Altogether, this study illustrate how a structured and sequential management may help to reduce neonatal sepsis mortality^4^. Benefit of biomarkers for EONS in LRS is questionable especially in regards to cost-effectiveness and decision-making. In settings with high burden of sepsis and LRS, the low specificity of biomarkers may significantly increase unnecessary treatment and therefore having low cost-effectiveness and risk of aggravating the development of multiresistant pathogens hospital ecology.

In this study we showed that cord blood PCT, and all tested biomarkers, are inferior to clinical criterion for neonatal sepsis diagnosis in Sub-Saharan African population with maternal fever at delivery being a key risk factor for EONS Expression of *CD74* was shown to be predictive of mortality in infants with neonatal sepsis. Kinetics of *CD74* expression following neonatal sepsis showed a prolonged impairment in survivors. In high-risk patients, *CD74*/IP-10 ratio was found to have a good accuracy for neonatal sepsis diagnosis, depicting a novel signature for defective Th1 response and altered antigen presentation seen in sepsis and sepsis-associated immune depression.

## Methods

### Study design and participants

Participants were delivered in two sub-urban health centres (sub-urban arm) and three urban University hospitals (hospital arm) in the Abomey-Calavi, Sô-Ava and Cotonou districts in the South Benin region where malaria is hyper-endemic^34^. In both arms, only infants born from mother living in the Abomey-Calavi district were recruited to facilitate the follow-up and minimize effect of geographical origins. In the sub-urban arm, that includes only normal geatation with low-risk delivery (no maternal risk factors for infection), all consecutive births were included, whereas in the hospital arm only newborns born from mothers with maternal-foetal risk factors for infection (prematurity, prolonged rupture of the membrane, maternal fever) were included. In both arms, the exclusion criteria included maternal HIV positive status, major congenital malformation and refusal of consent. All children from both arms were followed clinically on a bi-monthly base during the first 3 months of life. The follow-up consisted of scheduled home visits and unscheduled emergency visits if the infant was ill. The study protocol was approved by the Comité d’Ethique de la Recherche – Institut des Sciences Biomédicales Appliquées (CER-ISBA 85-5). Written informed consent was obtained from parents. All methods were performed in accordance with the relevant guidelines and regulations.

### Exposures and neonatal sepsis definition

The exposure were occurrence of gestational malaria (GM). GM was defined as a malaria infection during pregnancy or at delivery. For women in the suburban arm, malaria screening was performed at each scheduled prenatal visit using a thick blood smear. Mothers from the Hospital arm were screened only at the time of delivery. In this group, antenatal malaria was established on the basis of mother’s anamnesis. For both study arms, placental blood smear and mother’s peripheral blood smear were performed. The Lambaréné technique was used to quantify parasitaemia with a detection threshold of five parasites per microliter^34^. Neonatal sepsis was suspected in neonates with more than two of the following criteria being present: neutrophil count < 7500/mm^3^ or > 14 500/mm^3^, band form > 1500/mm^3^, immature/total neutrophils ratio > 0.16, platelets count < 150 000/mm^3^ and CRP > 10 mg/L. Suspected neonatal sepsis was considered as clinical sepsis when the following clinical signs were associated: temperature irregularity; respiratory distress or apnoea; seizures, altered tonus, irritability or lethargy; vomiting, altered feeding pattern, ileus; skin perfusion alteration, haemodynamic signs (tachycardia, hypotension); hypoglycaemic/hyperglycaemic, hyperlactatemia or identification of focal infection such as soft tissue infection or conjunctivitis^19,57,58^. All newborns with a clinical sepsis were subsequently adjudicated by one independent pediatrician (PT) and sorted into ‘presumed sepsis’ and ‘definite clinical sepsis’ grouped as “adjudicated sepsis”. In discordant cases, a second independent pediatrician (ULT) performed the final adjudication with access, in addition to the full medical file review, to microbiological cultures results. Parallel to microbiological culture (BACT/ALERT® system), specific BioFire® FilmArray® panels (bioMerieux, Marcy-l’Etoile, France) were run for all positive blood cultures (Blood Culture Identification (BCID) panel), cerebrospinal fluids (meningitis/encephalitis panel), respiratory and gastrointestinal samples (Pneumonia and Gastro-Intestinal panels). All studied biomarkers were kept blinded for the adjudication.

### Biomarkers sampling

At birth and at follow-up visits, the clinical examination data of the children were collected. Blood samples were obtained at birth, then at week (W)1, W4, W8 and W12. The study protocol has been described in detail elsewhere^34^. In brief, 18 mL cord blood at birth and 2 mL peripheral blood were collected in PAXgene™ Blood RNA tubes (PreAnalytix, Hilden, Germany) for transcriptomic biomarkers study. For protein biomarkers study 9 mL cord blood at birth and 500 µL to 1 mL peripheral blood were collected in heparin tubes. PAXgene™ Blood samples were stabilized at least 2 h at room temperature after collection and frozen at − 80 °C following the manufacturer’s guidelines. Heparin blood samples were centrifuged at 1500 to 2000 rpm for 5 min. The plasma obtained was stored at − 80 °C.

### RNA extraction, reverse transcription and quantitative PCR

Total RNA was extracted from cord blood by QIAsymphony SP/AS (QIAGEN Hilden, Germany) using PAXgeneTM Blood RNA Kit (PreAnalytix, Hilden, Germany) according manufacturer guidelines. The RNA integrity was measured prior to RNA amplification using a Bioanalyser 2100 (Agilent Technologies, Palo Alto, CA) in accordance with the manufacturer’s instructions (RNA integrity number ≤ 6 were excluded [min 6.5–max 9.4]). The expression level of *CX3CR1* and *CD74* was measured by RT-qPCR using ABI7500 thermocycler (Applied BioSystems®, California,USA) from 10 ng of RNA samples using prototype Argene® kit (bioMerieux, France) following the manufacturer's instructions. The determination of the copy concentration of each gene is performed using a calibration curve. The results are expressed as the ratio of the concentrations of *CX3CR1* or *CD74* to those of *HPRT1* (Hypoxanthine Phosphoribosyltransferase 1) used to normalize the gene expression results.

### Protein quantification

PCT, IL-6, IL-10 and IP-10 proteins in plasma were analyzed in a batch by Simplex AssaysTM according to the manufacturer’s instructions as previously described^59^. The Ella microfluidic analyzer (Protein Simple, San Jose, CA, USA) was used to assess cytokine concentrations^60^.

### Outcomes

The primary outcome was the diagnosis of clinical neonatal sepsis, and secondary outcome was mortality within the first 3 months of life. Neonatal sepsis diagnosis was established by the local paediatrician based on the clinical examination of the child and initial laboratory workup including haemogram, C-reactive protein (CRP) and microbiological cultures (blood, cerebral fluids and urine). Neonatal sepsis that occurred within the first 72 h following birth was considered as an early onset neonatal sepsis (EONS), and late onset (LONS) thereafter (for detailed algorithm for sepsis diagnosis, see published study protocol^34^).

### Statistical analysis

An independent statistician (FB) (Soladis Inc. Lyon, France; https://www.soladis.com) and IRD biostatistician (GA) supported the statistical methodology and performed all analysis. Statistical analyses were performed using R software version 3.6.1. The variables were assessed for normality using Kolmogorov Smirnov test. Numbers and frequency were used for qualitative data and medians and IQR (inter-quartile range: [Q1–Q3]) for quantitative data. Qualitative variables were compared using the Chi-squared test (or Fisher’s exact test for small expected numbers). The distribution of quantitative data was compared using Student’s t-test (or the Mann–Whitney t-test when distribution was not normal or Welch test when homoscedasticity was rejected) if 2 groups were compared. If more than 2 groups, the distribution of quantitative data was compared using Anova test (or the Kruskal–Wallis test when distribution was not normal or when homoscedasticity was rejected).

To evaluate their diagnostic accuracy, data-driven analysis was performed. Three models were used:

Model 1 encompassed only Hospital patients either with or without clinical sepsis; Model 2 enriched the non-sepsis group with unseptic patient from the suburban arm. Model 3 refined the sepsis group with only the culture positive sepsis considered. Selection of cut-off values or discrimination values defining the positive and negative test results were performed. Several methods for selecting optimal cut-off values in diagnostic tests are proposed in the literature depending on the underlying reason for this choice. Here, we selected a cut-off to have a sensitivity of 0.95 and maximize specificity. This choice of cut-off was the same in the rest of the publication^61^. CD74/IP-10 was the score corresponding to the division of CD74 gene expression level by IP-10 serum concentration. We used three datasets to test more complex models, either genes (noted CX3CR1 & CD74), proteins (noted Protein biomarkers) or sets of biomarkers (noted All biomarkers). To avoid overfitting and to compare our models, we used random sampling which takes place within each class and must preserve the overall distribution of data by class. To do this, we created a distribution, repeated 200 times, of 75/25% of the data. This distribution was used to optimize hyperparameters with package caret. Then we compared the average AUC and select the best average AUC for each dataset between all models. AUC accuracies were compared using Bootstrap approach. A *p* value < 0.05 was considered as significant.

For comparison between clinical variables (VC) and biomarkers, we selected the clinical variables of interest following an expert opinion (PT) and corresponding to neonatal risk factors (eg. Gestational age, weight, maternal risk factors, multiple gestation, APGAR score) (Supplementary Table 1). In order to compare clinical and biomarker data, we transformed the data into the same referential to be able to compare them. We transformed the categorical clinical variables into a complete set of dummy variables^62^. Different transformations were then applied to the data set. A Yeo-Johnson transform is a non-linear transformation that reduces skewness and approximates a normal law. We centered (subtracts the mean of the variable’s data) and scaled data (divides the standard deviation).

For the establishment of heatmap, Partial Last Squares (PLS) Regression was used to compare the two datasets. This algorithm comes from the mixOmics package. Biomarkers were deflated with respect to the information extracted/modelled from the local regression on Clinical Variables. Consequently, the latent variables computed to predict Biomarkers from Clinical Variables are different from those computed to predict Clinical Variables from Biomarkers. One matrix Clustered Image Map (CIM) is a 2-dimensional visualization with rows and/or columns reordered according to some hierarchical clustering method to identify interesting patterns. The CIM allows to visualize correlations between variables. Generated dendrograms from clustering were added to the left side and to the top of the image. The used clustering method for rows and columns is the complete linkage method and the used distance measure is the distance Euclidean. We showed only variables with co-variances greater than max(covariance)/2^62^.

To study clinical items and biomarkers factors associated with clinical sepsis, and death, we used univariate and multivariate logistic regression models. Three separate models were performed, one for clinical sepsis (Yes/No), the second for confirmed sepsis, corresponding to adjudicated sepsis (Yes/No) (data not shown) and a last one for the death (Yes/No). All variables with a p-value below 0.25 in univariate analysis were selected for the multivariate analysis. In addition, all biomarkers were forced into the multivariate models. Then, a manual backward selection procedure was used to obtain the final adjusted multivariate model, a *p*-value of < 0.05 was considered statistically significant. Stata version 15 for Windows (Stata Corp., College Station, TX) was used for statistical analyses.

### Statistical algorithms

We tested several algorithms: Bagged CART (package ipred), CART (package rpart), Naive Bayes (package klaR and naivebayes), Multi-Step Adaptive MCP-Net (package msaenet), Bagged Flexible Discriminant Analysis and Flexible Discriminant Analysis (package earth), Multi-Layer Perceptron (package RSNNS), Regularized Random Forest (package RRF), Quadratic Discriminant Analysis and Linear Discriminant Analysis (package MASS), Robust Linear Discriminant Analysis (package rrcov), Robust Mixture Discriminant Analysis (package robustDA), High Dimensional Discriminant Analysis (package HDclassif), Random Forest (package randomForest and ranger), Stochastic Gradient Boosting (package gbm), Conditional Inference Tree (package party), Neural Network and Neural Networks with Feature Extraction (package nnet), Generalized Additive Model using Splines (package mgcv), Distance Weighted Discrimination with Polynomial Kernel and Distance Weighted Discrimination with Radial Basis Function Kernel (package kerndwd), Support Vector Machines with Radial Basis Function Kernel (package kernlab), Single C5.0 Ruleset (package C50), Boosted Logistic Regression (package caTools), Oblique Random Forest (package obliqueRF), Sparse Partial Least Squares (package mixOmics), k-Nearest Neighbors.

For the outcome “sepsis adjudicated”, the model Multi-Layer Perceptron was selected for “All biomarkers”, the model Quadratic Discriminant Analysis was selected for “Protein Biomarkers” and k-Nearest Neighbors for “CX3CR1 & CD74”. For the outcome “clinical sepsis”, the model Generalized Additive Model using Splines was selected for “All biomarkers”, the model Distance Weighted Discrimination with Polynomial Kernel was selected for “Protein Biomarkers” and Support Vector Machines with Radial Basis Function Kernel for “CX3CR1 & CD74”. For the outcome death, the model Sparse Partial Least Squares was selected for “All biomarkers”, the model Sparse Partial Least Squares was selected for “Protein Biomarkers” and Neural Network for “CX3CR1 & CD74”. The random forest model did not select any variables (6Biom + 16 VC). The Stochastic Gradient Boosting model selected “5 Biomarkers and 6 Clinical Variables”. The Flexible Discriminant Analysis model selected “3 Biomarkers and 4 Clinical Variables”. The Multi-Step Adaptive MCP-Net model selected “1 biomarker and 2 Clinical Variables”. See clinical variables legend in Supplementary Table 1.

### Adaptive

The study protocol was approved by the local institutional review board (Comité d’Ethique de la Recherche de l’Institut des Sciences Biomédicales Appliquées CER-ISBA 85-5). Written informed consent was obtained from parents.

## Supplementary Information

Supplementary Table 1.

## Data Availability

The datasets used and/or analysed during the current study available from the corresponding author on reasonable request.

## Abbreviations

CD74: Cluster of differentiation 74
CX3CR1: C-X3-C motif chemokine receptor 1
PCT: Procalcitonin
IL-6/-10: Interleukin-6/-10
IP-10: Interferon inducible protein 10
CRP: C reactive protein
LMIC: Low- and middle-income country
SSA: Sub-Saharan Africa
GM: Gestational malaria
E-/L-ONS: Early-/late-onset neonatal sepsis
W: Week
AUC: Area under the curve
GA: Gestational age
mHLA-DR: Monocyte Human leukocyte antigen-DR
