# Supplementary material for: Prospective multicentre study of host response signatures in neonatal sepsis in Sub Saharan Africa
Source: Sci Rep. 2022 Dec 12;12:21458. doi: 10.1038/s41598-022-25892-x (PMC9743113; doi:10.1038/s41598-022-25892-x)
Supplement: Supplementary file 1 — Supplementary Table 1. [file 41598_2022_25892_MOESM1_ESM.docx]

**Supplementary Table 1 : Cutoff values of biomarkers for diagnosis or prognostic of neonatal clinical sepsis**

|  | **MODEL 1*** | | | **MODEL 2**** | | | **MODEL 3***** | | |
| --- | --- | --- | --- | --- | --- | --- | --- | --- | --- |
|  | **Cutoff** | **Se** | **Sp** | **Cutoff** | **Se** | **Sp** | **Cutoff** | **Se** | **Sp** |
| *CD74 ^¶^* | 12,24 | 0,96 | 0,05 | 14,15 | 0,96 | 0,08 | 15,64 | 0,98 | 0,05 |
| *CX3CR1 ^¶^* | 13,55 | 0,97 | 0,02 | 5,66 | 0,96 | 0,03 | 5,66 | 0,95 | 0,02 |
| IL-10 (pg/ml) | 2,7 | 0,96 | 0,02 | 1,98 | 0,96 | 0,04 | 1,98 | 0,98 | 0,04 |
| IL-6 (pg/ml) | 1,44 | 0,98 | 0,02 | 2,06 | 0,95 | 0,08 | 2,82 | 0,96 | 0,19 |
| IP-10 (pg/ml) | 416,9 | 0,96 | 0,07 | 298,11 | 0,96 | 0,10 | 35,93 | 0,96 | 0,06 |
| PCT (pg/ml) | 401,8 | 0,96 | 0,06 | 168,50 | 0,95 | 0,08 | 223,60 | 0,96 | 0,11 |
|  | **MODEL 1^a^** | | | **MODEL 2 ^b^** | | | **MODEL 3 ^c^** | | |
|  | **Cutoff** | **Se** | **Sp** | **Cutoff** | **Se** | **Sp** | **Cutoff** | **Se** | **Sp** |
| *CD74 ^¶^* | 9,47 | 0,95 | 0,10 | 12,37 | 0,97 | 0,09 | 6,36 | 1,00 | 0,36 |
| *CX3CR1^¶^* | 1,71 | 0,95 | 0,06 | 5,98 | 0,97 | 0,02 | 13,55 | 1,00 | 0,01 |
| IL-10 (pg/ml) | 3,75 | 0,95 | 0,50 | 2,11 | 0,95 | 0,07 | 3,07 | 1,00 | 0,28 |
| IL-6 (pg/ml) | 22,37 | 0,95 | 0,34 | 2,20 | 0,97 | 0,10 | 4,64 | 1,00 | 0,27 |
| IP-10 (pg/ml) | 44,29 | 0,95 | 0,05 | 35,93 | 0,95 | 0,07 | 47,92 | 1,00 | 0,19 |
| PCT (pg/ml) | 214,83 | 0,95 | 0,05 | 195,44 | 0,95 | 0,09 | 289,29 | 1,00 | 0,19 |

***** Model 1 : Hospital arm sepsis (n=163) **vs** hospital arm no sepsis (n=257)

****** Model 2 : Hospital arm sepsis (n=163) **vs** no sepsis (suburban arm n=47 & hospital arm n=245)

******* Model 3: Hospital arm sepsis with positive blood culture (n=55) **vs** no sepsis (suburban arm n=47 & hospital arm non sepsis n=245)

^a^ Hospital arm -Survivors (n=372) vs Hospital arm -sepsis non survivors (n=48)

^b^ Survivors suburban arm (n=47) + Hospital arm sepsis (n=368)) vs Hospital arm -sepsis non survivors (n=47)

^c^ Survivors (n=399) vs Hospital arm - positive blood culture sepsis non survivors ( n=10)

Cutoff values were selected to have a minimal of 0,95 sensibility with a maximum specificity

*CD74*, HLA class II histocompatibility antigen gamma chain; *CX3CR1;* CX3C chemokine receptor 1; *IL*, Interleukin; PCT, procalcitonin.

*^¶^ CD74* and *CX3CR1* are expressed as relative expression to *HPRT1.*
